# An open-access database of video stimuli for action observation research in neuroimaging settings: psychometric evaluation and motion characterization

**DOI:** 10.3389/fpsyg.2024.1407458

**Published:** 2024-09-25

**Authors:** Christian Georgiev, Thomas Legrand, Scott J. Mongold, Manoa Fiedler-Valenta, Frédéric Guittard, Mathieu Bourguignon

**Affiliations:** ^1^Laboratory of Neurophysiology and Movement Biomechanics, UNI – ULB Neuroscience Institute, Université Libre de Bruxelles (ULB), Brussels, Belgium; ^2^Laboratoire de Neuroanatomie et Neuroimagerie Translationnelles, UNI – ULB Neuroscience Institute, Université Libre de Bruxelles (ULB), Brussels, Belgium; ^3^BCBL, Basque Center on Cognition, Brain and Language, Donostia-San Sebastian, Spain

**Keywords:** videos, database, action observation, psychometrics, movement onset detection, neuroimaging

## Abstract

Video presentation has become ubiquitous in paradigms investigating the neural and behavioral responses to observed actions. In spite of the great interest in uncovering the processing of observed bodily movements and actions in neuroscience and cognitive science, at present, no standardized set of video stimuli for action observation research in neuroimaging settings exists. To facilitate future action observation research, we developed an open-access database of 135 high-definition videos of a male actor performing object-oriented actions. Actions from 3 categories: kinematically natural and goal-intact (*Normal*), kinematically unnatural and goal-intact (*How*), or kinematically natural and goal-violating (*What*), directed toward 15 different objects were filmed from 3 angles. Psychometric evaluation of the database revealed high video recognition accuracy (*Mean* accuracy = 88.61 %) and substantial inter-rater agreement (Fleiss' *Kappa* = 0.702), establishing excellent validity and reliability. Videos' exact timing of motion onset was identified using a custom motion detection frame-differencing procedure. Based on its outcome, the videos were edited to assure that motion begins at the second frame of each video. The videos' timing of category recognition was also identified using a novel behavioral up-down staircase procedure. The identified timings can be incorporated in future experimental designs to counteract jittered stimulus onsets, thus vastly improving the sensitivity of neuroimaging experiments. All videos, their psychometric evaluations, and the timing of their frame of category recognition, as well as our custom programs for performing these evaluations on our, or on other similar video databases, are available at the Open Science Framework (https://osf.io/zexc4/).

## Introduction

In the past two decades, action observation research has been facilitated by technological advancements which allow researchers to easily record and present videos of biological motion. This in turn allowed for the use of video stimuli in controlled neuroimaging and psychophysics investigations of the neural processing of the movements which make up human naturalistic action. Consequently, experimental paradigms involving presentation of pre-recorded videos of various types of bodily motion have become ubiquitous in neuroscience, cognitive science, and psychology. Such investigations have produced great advances in the understanding of biobehavioral phenomena ranging from the cellular to the cognitive level, such as the activity of mirror neurons (Arnstein et al., 2011; Braadbaart et al., 2013; Moriguchi et al., 2009), the Mu and Beta oscillations of the sensorimotor cortex (Avanzini et al., 2012; Brunsdon et al., 2019; Muthukumaraswamy and Johnson, 2004; Quandt and Marshall, 2014), motor affordances (Bach et al., 2011; Tipper et al., 2006), action identification and discrimination (Orban et al., 2019; Urgen and Orban, 2021; Vannuscorps and Caramazza, 2016), gesture recognition (Beattie and Shovelton, 2002), observation learning (Buccino et al., 2004; Malfait et al., 2010), theory of mind (Caillaud et al., 2020; Saxe et al., 2004; Sylwester et al., 2012), and empathy (Rosenthal-von der Pütten et al., 2014; Tholen et al., 2020). To address these phenomena, typically, videos of an agent performing a manual movement are presented to observers either in blocked or in pseudo-random counterbalanced designs while electrophysiological or hemodynamic measures of neural activity are recorded. This approach allows for a resolute assessment of the neurocognitive processing of observed actions, carried out by bottom-up perceptual as well as top-down cognitive neuronal mechanisms. Thus, the approach allows for the investigation of the underpinnings of perception, comprehension, and acquisition of complex action and intention in both neurotypical (Biagi et al., 2016) and neurodivergent (Scott et al., 2020; Spengler et al., 2010) populations.

To effectively capture the neural correlates of the detection and comprehension of the nuanced movements which comprise complex naturalistic action, however, the presented video stimuli must be constructed in accordance with the highest psychometric standards. Duration, frame rate, pixel resolution, color palette, luminosity, filming angle, depicted body segments, gender and handedness of the actor, familiarity with the action and the actor, and recognizability of the action have all been controlled for, at least to a certain extent, in many individual action observation studies. Yet, across studies, there is substantial heterogeneity in the presented video stimuli, with some authors presenting videos depicting only the hand, others presenting videos depicting the entire upper body of the actor; some authors presenting videos from a first-person perspective, others from a third-person perspective; and some authors presenting videos in black and white, others in color. With the advancement of video recording equipment, the overall quality of the video stimuli increases and novel tools have been developed for monitoring changes across video recordings, including nuanced changes in posture (Zouba et al., 2008) or motion kinematics (Trettenbrein and Zaccarella, 2021) of the actor across videos. Still, standardized sets of high-quality stimuli, made specifically for action observation research in neuroimaging settings, which could be used across experiments and laboratories, are scarce, and most investigators in the field construct their own stimuli or rely on non-dedicated freely-available videos from the internet. The reliance on such stimuli which likely differ in either technical properties (e.g., pixel resolution and frame rate) or higher-order characteristics of motion (e.g., goal-directedness and intention relation) prevents the comparison of results across experiments, complicates the conduction of robust and reliable meta-analyses, and hence negatively impacts the reproducibility of action observation research. More recently, open-access multipurpose databases of video stimuli have been developed (Cipriano et al., 2023; Di Crosta et al., 2020; Umla-Runge et al., 2012; Urgen et al., 2022), however, videos therein feature only kinematically correct natural actions, which does not allow for investigations of observation and comprehension of bodily motion with a varying degree of kinematic and goal accuracy. In addition, in spite of the high quality of the videos in these databases, the motion depicted therein was not characterized in terms of when within the video the action begins and when a human observer would actually recognize the action as intended by the experimenter. The absence of a marker indicating when the desired evoked electrophysiological or hemodynamic neural response is likely to occur complicates the use of these databases in neuroimaging settings.

An initial challenge of the serial presentation of a large number of videos in neuroimaging settings is that differences in the timing of movement onset can exist across videos, even among the videos of a carefully constructed stimulus set where all videos are controlled for duration and frame rate. Although some authors have identified and reported the timing of motion onset of their stimuli (e.g., Platonov and Orban, 2016), this practice is often overlooked or underreported in the literature. This is especially an issue for electrophysiological neuroimaging experiments since these typically require averaging of neural responses evoked by a large number of videos (Huettel, 2012), and both time domain and time-frequency domain analyses have to be precisely timed to video onset. In that setting, seemingly small differences in the actual visual detection of movement across videos are likely to contaminate the contrasts between the experimental conditions. For instance, if an onset trigger is sent at the very first frame of each video stimulus' presentation, a discrepancy in movement onset across videos of as little as 3 frames at 60 Hz translates to a jitter of 50 ms. Such jitter can substantially impact the shape and timing of the observed evoked neural responses, resulting in noisier and flattened out averaged neural responses and hence, difficulties in detecting differences between experimental conditions. In hemodynamic neuroimaging, the negative impact of discrepancies between video onset and actual movement onset also exists, although it may be smaller due to the slow nature of the blood-oxygen-level-dependent (BOLD) signal.

In an analogous fashion, the issue of timing and triggering also concerns the timing of the actual conscious recognition of the higher-order properties of movement, such as the degree of integrity of movement kinematics, goal, or intention across different videos, which are the central manipulation of numerous studies addressing the neural and cognitive representations of normal and abnormal movement (e.g., Stapel et al., 2010; Cheng et al., 2017). Such studies often imply presentation of videos that either depict natural or unnatural actions with varying degrees of goal integrity. The videos of natural actions typically comprise kinematically correct movements, as would normally be performed and observed in everyday settings, whereas the videos of unnatural actions comprise kinematically incorrect odd movements, which are unlikely to be performed or observed in everyday settings. However, the motor redundancy and abundance of any complex human action (Gera et al., 2010; Steen et al., 2011) suggest that it is highly likely that the kinematic nature and goal integrity of the actions in different videos are recognized at very different time points. Once again, averaging neural responses with jittered onsets will impact the shape and timing of the observed evoked neural responses and hence, severely contaminate any contrast between natural and unnatural, and between goal-intact and goal-violating action categories.

Therefore, determining when within a video stimulus movement onset and kinematic and goal category are actually perceived is crucial for effective experimental design. Although precise motion characterization of videos can be obtained with complex artificial intelligence computer vision algorithms (Vrigkas et al., 2015), such algorithms are not always freely available and require advanced expertise in deep learning. With respect to motion onset detection, more user-friendly motion detection systems based on the frame-differencing method have been widely applied on video recordings within the field of counseling psychology (Paxton and Dale, 2013; Ramseyer and Tschacher, 2011), however, no such methodology has yet been applied for characterizing videos used as stimuli in action observation research. We propose that such a system can successfully be applied for identifying the first frame of motion onset within video stimuli, so that all video stimuli can subsequently be edited to start exactly at the identified frames. With respect to the recognition of higher-level properties of categories of videos with different kinematics and goal-integrity, no accessible and user-friendly systematic stimulus evaluation procedure exists. We propose that a straightforward psychophysics approach based on the classic up-down staircase methods (Levitt, 1971; Kaernbach, 1991; Wetherill and Levitt, 1965) can be applied for identifying the first frame at which a video can be categorized (i.e., recognition of the movement as kinematically correct or incorrect and goal-intact or goal-violating). Namely, if a video is played up until a given number of frames, then paused, and an observer is asked whether the motion depicted in the video was kinematically correct or not, and goal-intact or not, their response can be used for adjusting the number of frames played on the subsequent presentation of the same video to another observer. With enough between-subject iterations, an asymmetric staircase procedure governed by two simple rules: (1) If the category of motion has been correctly detected within the played segment, the number of played frames decreases on the subsequent presentation and (2) If the category of motion has not been detected within the played segment, the number of played frames increases on the subsequent presentation; should converge on the frame where the category of the video becomes discernible for naïve human observers. The identified frames can be used for implementing precise triggering for the identification of brain or behavioral responses to action kinematics and goal integrity.

Aspiring to improve the feasibility of action observation research, we introduce a large psychometrically evaluated open-access database of videos tailored to the demands of neuroimaging experiments. More precisely, we present 135 videos depicting a goal-directed action that is either kinematically correct or not, and goal-intact or not, filmed from a third-person perspective from 3 angles (portrait, left profile, and top). These videos should be useful for future investigations of the neural mechanisms of action and goal perception and comprehension. Moreover, we provide an easy to implement, open-source frame-differencing motion detection system for identifying the frame of motion onset within each video and a straightforward procedure for editing the videos based on their motion onset. We also provide a straightforward open-source up-down staircase procedure for identifying the frame on which kinematics and goal-integrity are recognized within each video. Both procedures are purposefully designed in a way that would allow any reader to easily implement them on our, or another set of similar video stimuli that depict motion of a single agent.

## Methods

### Participants

Fifty-one adults (27 Female, *Mean* ± *SD* age: 28.47 ± 7.16 years) provided ratings for psychometric evaluation of the videos. A different sample of 17 adults (8 Female, *Mean* ± *SD* age: 25.82 ± 4.6 years) participated in the up-down staircase category recognition procedure. Participants were recruited from the Université Libre de Bruxelles (Brussels, Belgium). All participants were healthy, with no known neurological or psychiatric disorders, had normal or corrected-to-normal vision, and gave their written consent for participation in the study. The protocols were approved by the local ethics committee and the study was conducted in accordance with the Declaration of Helsinki.

### Stimuli

**Figure 1:**
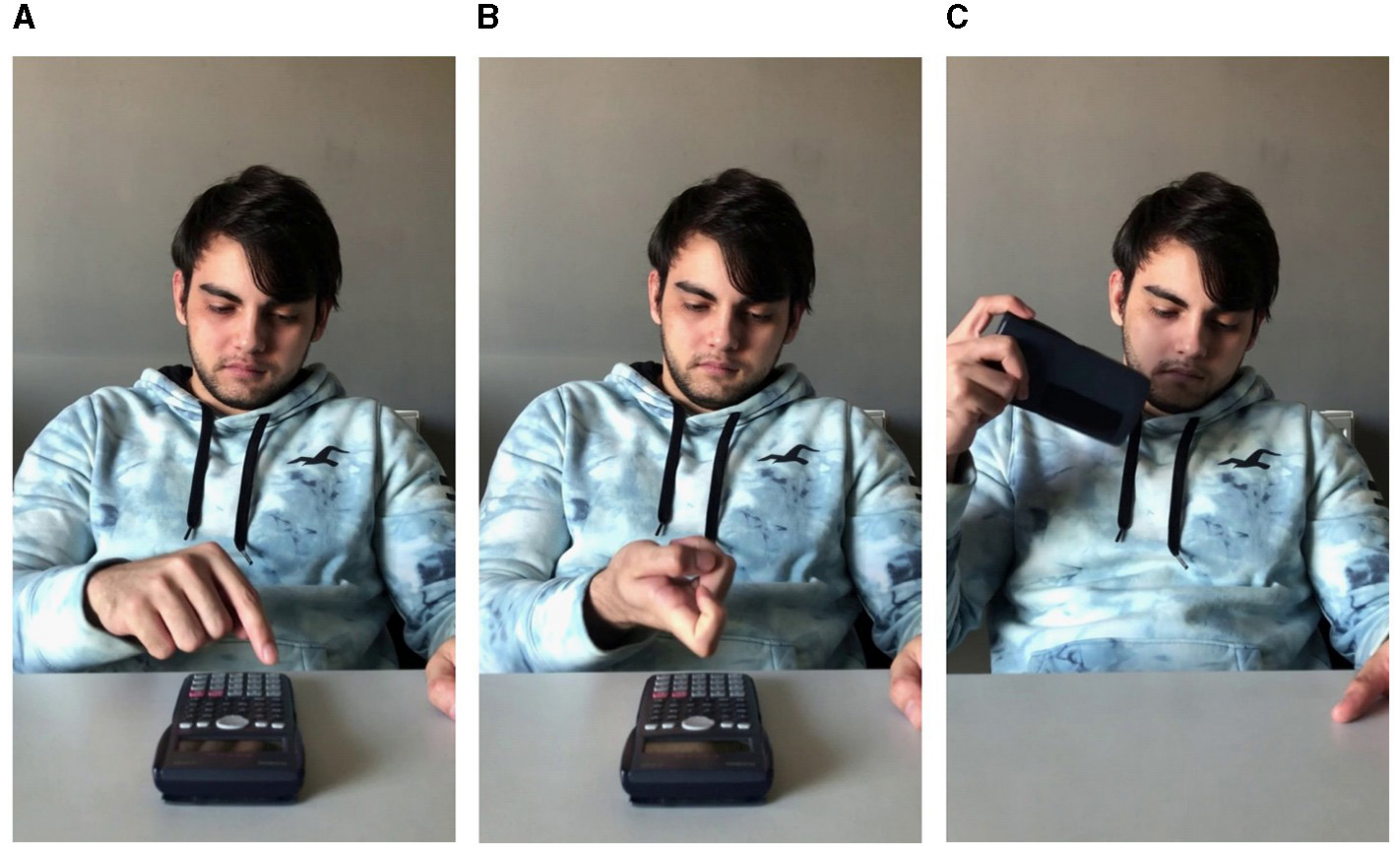
Representative frames extracted from a video depicting a *Normal* **(A)**, *How* **(B)**, and *What* **(C)** use of a calculator.

**Figure 2:**
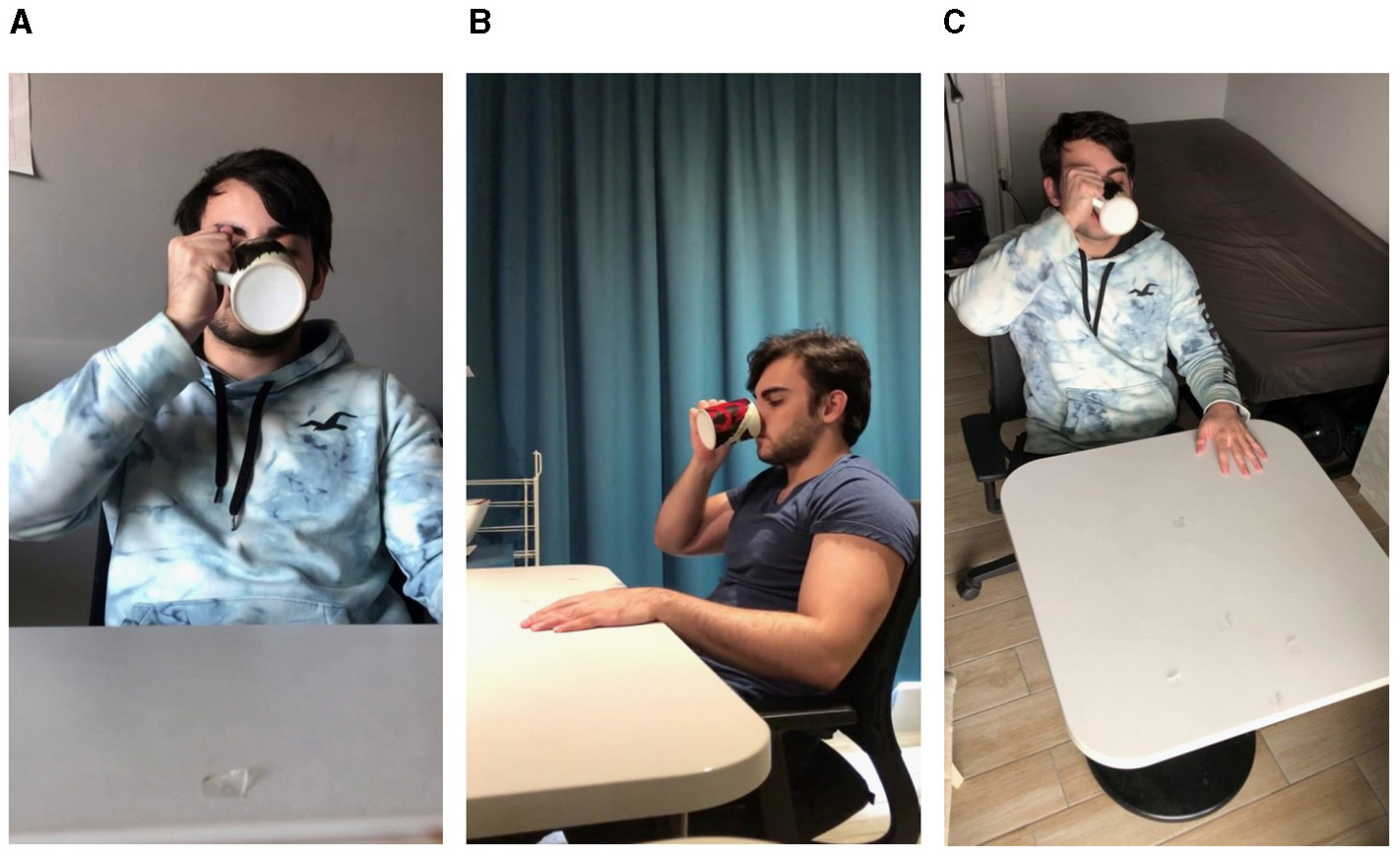
Representative frames extracted from videos depicting a *Normal* use of a cup from a portrait **(A)**, profile **(B)**, and a top angle **(C)**.

A database of videos was created specifically for the demands of subsequent action observation experiments. The database consists of high-resolution video recordings filmed in 4K (at 60 frames/second with a 12-megapixel Apple iPhone 12 camera in portrait orientation, positioned on a stationary tripod) and subsequently resized to 720 × 1,280 pixels. The videos depict a male actor (Caucasian, Age = 22 years) seated in front of a table and performing a goal-directed action with a common easy to manipulate everyday object (calculator; cap; coffee jar; comb; computer mouse; cup; fork; glasses; hat; headphones; hourglass; pen; pencil case; ruler; scissors). Each object from this set is expected to be highly familiar to human observers and the kinematics of past manual interactions with each object (and therefore the motor affordances recalled at the sight of the object) are expected to show a high degree of convergence across participants. Hence at action observation, neuroimaging results are less likely to be contaminated by inability to recognize the target object or the kinematics and goals of the performed actions. With respect to the kinematic and goal integrity, actions from 3 categories were filmed: *Normal* (i.e., the object was used in the most kinematically natural way and for its intended goal; e.g., a calculator was placed on the table and typed on with the tip of the index finger; Figure 1A), *How* (i.e., the object was used in a kinematically unnatural way and for its intended goal; e.g., a calculator was placed on the table and typed on with the knuckle of the index finger; Figure 1B), and *What* (i.e., the object was used in a kinematically natural way but not for its intended goal; e.g., a calculator was lifted off the table and used as a fan; Figure 1C). The three categories of actions were conceptualized such that they would allow for electrophysiological or hemodynamic contrast experiments isolating the neural processing of action kinematics (*Normal—How*) and action goals (*Normal—What*). Inspired by Iacoboni et al. (2005) and following the recommendations of Sonkusare et al. (2019), all actions were filmed in living settings, providing a static naturalistic background and hence increasing the ecological validity of the stimuli. Each action was filmed from a third-person point of view from 3 angles: portrait, left profile, and top (Figure 2), corresponding to the typical perspectives from which actions of a conspecific are observed in daily life and allowing investigators to better define the extent to which an observer's perspective affects action recognition, action embodiment and affordance recall. In total, 15 actions were filmed in each category and from each filming angle resulting in 135 videos, each with a duration of 3–6 s. All videos in .m4v and .mat format can be found in folder “Video Database” at the Open Science Framework: https://osf.io/zexc4.

### Psychometric evaluation

The participants were instructed that they would be asked to evaluate a number of videos in terms of their kinematic and goal integrity category. The three categories of videos, along with their labels (*Normal, How*, or *What*) were explained to the participants. *Normal* videos were defined as: “… actions depicting the natural and correct use of an object for its intended purpose in normal everyday settings”, the *How* videos were defined as: “… actions depicting an unnatural use of the object for its intended purpose”, and the *What* videos were defined as: “… natural use of an object outside of its intended purpose in normal everyday settings”. After having received the instructions the participants were allowed to ask for further clarification if necessary. The participants were then seated in front of a 17-inch computer monitor and were sequentially presented with the 135 videos in a pseudo-random order (multiple presentations of the same video were not allowed). After the end of each video, participants were prompted to classify it as “*Normal*”, “*How*”, or “*What*” by pressing a corresponding key on the keyboard. The entire rating procedure was completed in a single session, with no time constraint.

### Identification of the frame of motion onset

**Figure 3:**
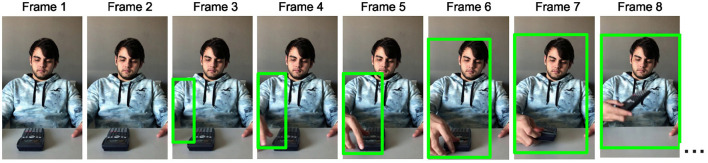
Schematic illustration of the operation of the motion detection system.

All videos were passed as input to a sensitive custom-made motion detection system based on the OpenCV library (Bradski and Kaehler, 2008) in Python 3 (Python Software Foundation, Wilmington, DE). This system was made such that it detected and reported the presence of motion per each frame of the input video object (Figure 3). To this end, each frame of a video was captured, converted to grayscale, and compared to the static initial frame of the respective video using a frame-differencing algorithm (Ramseyer, 2020).

Differences between the two frames were highlighted with a binary threshold method, setting pixels that were at least 10 % different on the grayscale to black and the rest to white. Subsequently, the contours of the resulting binary frame were extracted using a standard border following algorithm (Suzuki and Abe, 1985) and used to perform box detection of motion. To avoid detecting background noise as motion, a threshold on the minimal box area was set to 400 pixels and restricted in the Y dimension to the area corresponding to the location of the actor, thus assuring the detection of only meaningful biological motion of the actor. The first frame on which the system registered a difference between the two frames is reported as the frame of motion onset. For each video, the outcome of the frame-differencing procedure was inspected and compared to the frame of motion onset identified by a human observer (C. G.) during a frame-by-frame video presentation to assure accurate identification and reporting of each video's frame of motion onset. In case of disagreement, a correction was applied in favor of the judgment of the human observer. Video stimuli were then edited to start at the frame preceding that of motion onset. The Python scripts for performing the frame-differencing motion onset detection on this or any other similar set of videos and the script for breaking the videos into frames can be found in the folder “Motion Onset Frame-Tracing Procedure” at https://osf.io/zexc4/.

### Identification of the frame of category recognition

Following-up the general video recognition procedure, we implemented a novel approach for identifying the frame on which videos became discernibly recognizable as *Normal, How*, or *What* to human observers. The experimental settings and the instructions given to the participants were identical to the ones for the psychometric evaluation procedure, with the notable exception that participants were told that the video will pause at a certain time point and their task would be to infer whether it is “*Normal*”, “*How”*, or “*What”* to the best of their ability. Each participant completed a single iteration of an up-down staircase implemented by a custom-made MATLAB (Mathworks, Natick, MA) script. For each iteration, all videos were played once in a pseudo-random order, up to a specific frame. For the first iteration of the staircase, performed by the first participant, each video was played up to the frame corresponding to the 50th percentile of the video's frames and then paused. After each video's pause, the participant was prompted to report whether, based solely on the played video segment, they would classify the given video as “*Normal*”, “*How*”, or “*What*” via mouse click. On each subsequent iteration, performed by each subsequent participant, the number of frames that would be played from each video was adjusted based on the response of the participant in the previous iteration. A correct classification of a video resulted in a presentation of the given video with N frames less on the next iteration of the staircase, while a failure led to adding N frames more, where N decreased monotonously with the iteration number (n) following the formula N = Nframe/2 × 2-(n – 2)/2.5, where Nframe is the number of frames in the video. Iterations of the staircase were performed until N was smaller than 1 and hence the magnitude of up-down reversals became smaller than 1 frame, thus converging on that frame. The frame converged upon by the final iteration was taken as the frame on which the category (*Normal, How*, or *What*) becomes recognizable.

### Statistical analyses

R 4.1.1 (R Core Team, 2021) was used for all statistical analyses. Participants' ratings from the psychometric evaluation procedure were used for calculating validity (expressed as recognition accuracy in number and percentage of correctly classified videos) and inter-rater reliability (expressed as Fleiss' *Kappa*; Fleiss, 1971). We performed an exploratory analysis investigating differences in recognition accuracy of videos from different categories (*Normal, How*, and *What*) and filming angles (portrait, left profile, and top) with 2-way within-subjects ANOVA.

Following the up-down staircase procedure, we investigated potential undesirable systematic effects of categories (*Normal, How*, and *What*) and angles (portrait, left profile, and top) on motion onset and timing of classification of videos, respectively with separate 2-way ANOVAs. This was deemed necessary as, although general differences in video properties across videos are expected, systematic differences between different groups of videos need to be brought to light so that future researchers can take them into account when designing experiments.

For all within-subjects ANOVAs, a Greenhouse-Geisser correction was applied when the Mauchly test indicated a significant departure from sphericity. Significant interactions were further analyzed by breaking down the main ANOVA into one-way ANOVAs, and simple effects were followed by *post-hoc* Tukey pairwise comparisons. The R script for performing all analyses can be found in the folder “All Data and Analyses” at https://osf.io/zexc4/.

## Results

### Validity and reliability

**Figure 4:**
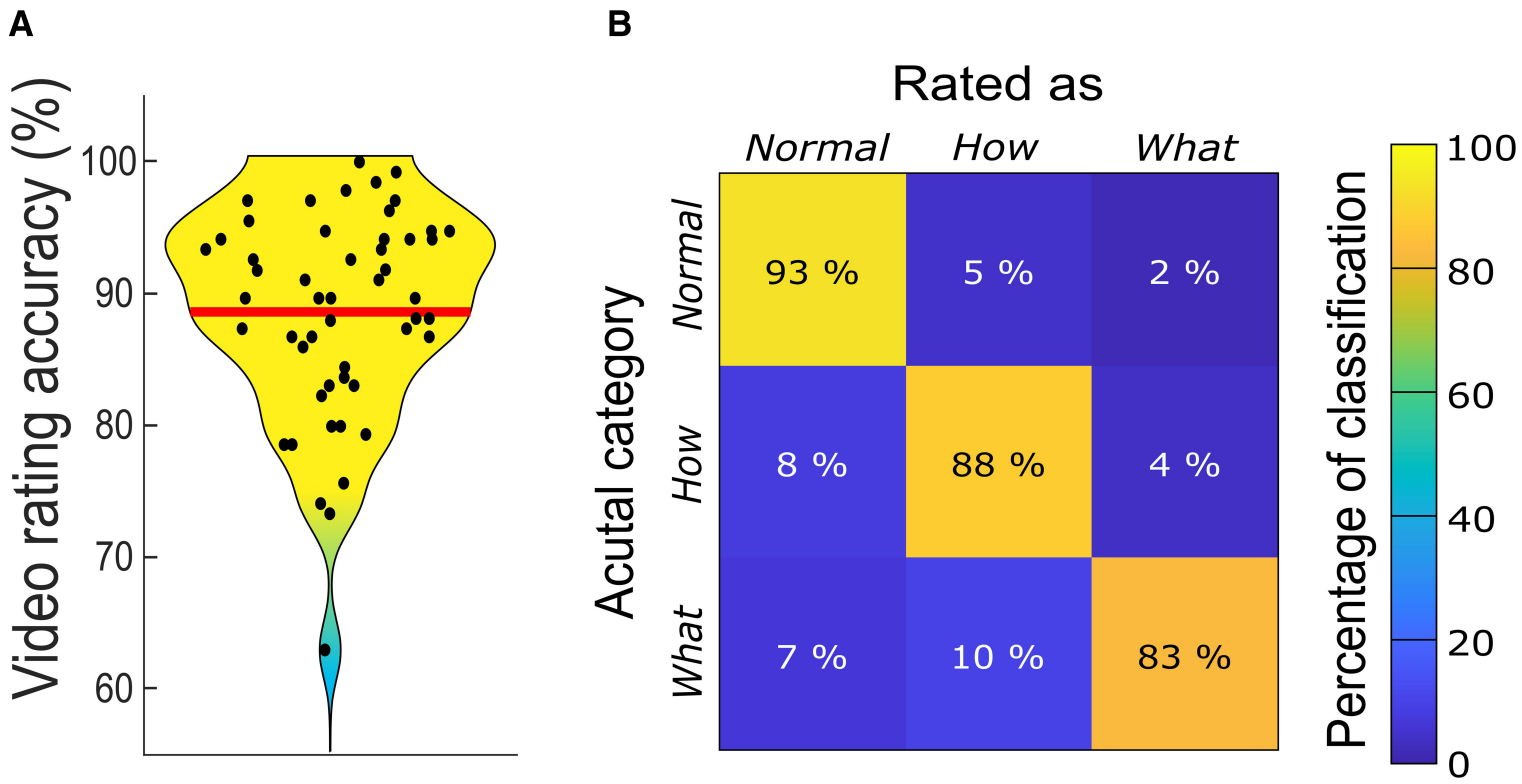
Video recognition accuracy of the whole sample **(A)** and recognition confusion matrix **(B)**. In **(A)** the red line indicates the mean recognition accuracy of 119.63 (88.61%) videos.

Figure 4A presents participants' overall category (*Normal, How*, or *What*) classification accuracy, which ranged between 85 and 135 videos (*Mean* = 119.63, *SD* = 10.41), corresponding to 63 % and 100 % accuracy respectively (*Mean* = 88.61 %, *SD* = 7.71 %). The average accuracy was significantly higher than that of 45 videos expected purely by chance (*t*_50_ = 51.18, *p* < 0.0001, 95% CI [116.69, 122.56], *d* = 7.17). Figure 4B presents the confusion matrix for classifications of *Normal, How*, and *What* videos. It indicates that most misclassifications involved reporting *How* or *What* videos as *Normal*, and *What* videos as *How*. Zooming in on these misclassifications by inspecting the confusion matrices for each video revealed that the differences are largely stimulus specific and stem from misclassifications of few individual videos. The confusion matrices for each video can be found in folder “All Data and Analyses” at https://osf.io/zexc4/. All in all, even for those select few videos the proportion of misclassification was, quite small compared to the proportion of correct classifications, therefore establishing good validity of the present dataset.

**Figure 5:**
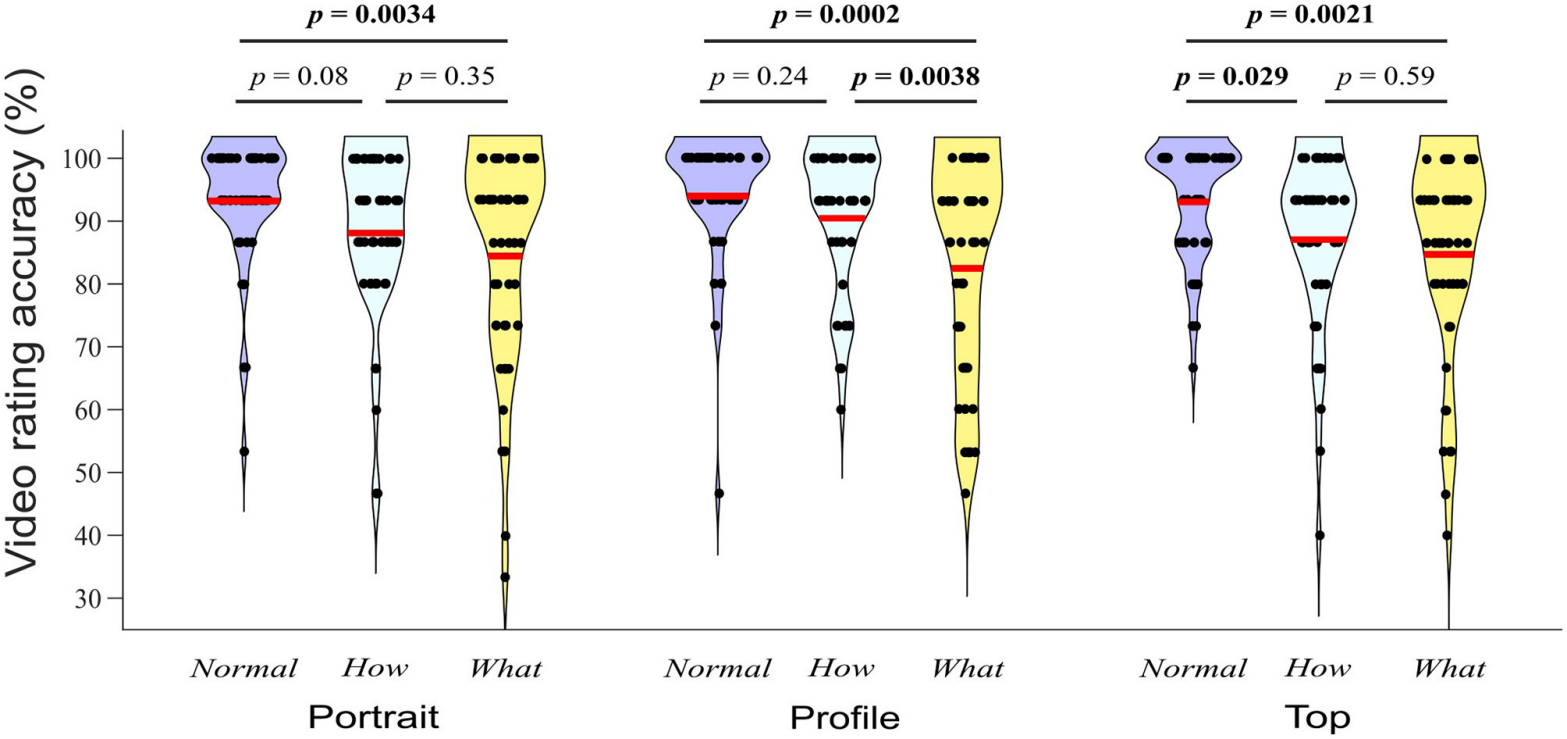
Distributions of the number of correctly recognized videos per category (*Normal, How*, and *What*) and filming angle (Portrait, Profile, and Top). Red lines indicate the respective means. *P*-values correspond to Turkey tests.

The ANOVA investigating the extent to which the classification accuracy depended on videos' category and filming angle revealed a significant effect of category (*F*_2, 98_ = 9.39, *p* < 0.001, partial η2 = 0.158), no effect of filming angle (*F*_2, 95_ = 0.55, *p* = 0.571, partial η^2^ = 0.011), and a significant category × filming angle interaction (*F*_4, 185_ = 3.08, *p* = 0.020, partial η^2^ = 0.058). To disentangle the interaction, we performed simple effects analyses of the factor category for each level of filming angle with one-way ANOVAs. These analyses revealed a significant effect of category on the classification accuracy for videos filmed from portrait angle (*F*_2, 98_ = 6.23, *p* = 0.003, η^2^ = 0.111), profile angle (*F*_2, 94_ = 12.11, *p* < 0.001, η^2^ = 0.195), and top angle (*F*_2, 100_ = 6.83, *p* = 0.002, η^2^ = 0.120). Figure 5 presents the outcome of *post-hoc* Tukey tests for each filming angle. A significant difference was found between *Normal* and *What* videos for all 3 angles, between *Normal* and *How* videos only within the top angle, and between *How* and *What* videos only within the profile angle. Considering all angles together, classification accuracy was marginally higher for *Normal* compared to *How* (*t*_50_ = 2.39, *p* = 0.052), significantly higher for *Normal* compared to *What* (*t*_50_ = 4.08, *p* = 0.0005), and marginally higher for *How* compared to *What* (*t*_50_ = 2.1, *p* = 0.099) videos. Although some statistically significant differences were found, it should be noted that values of classification accuracy were very high across all categories and angles, with mean accuracy ranging from 12.37 out of 15 for the *What* videos in profile view to 14.09 out of 15 for the *Normal* videos in profile view.

### Motion onset detection

As expected, even with the strict detection parameters we specified, the motion detection system reported differences in the onset of motion across the videos, with motion onset detected in the range 2–18 frames (*Mean* = 2.5, *SD* = 1.79), corresponding to time 0.03–0.3 s (*Mean* = 0.04 s, *SD* = 0.03 s). The 2-way ANOVA investigating for differences in motion onset between videos from different category and filming angle revealed no effect of category (*F*_2, 126_ = 0.54, *p* = 0.583, partial η^2^ = 0.009), a significant effect of filming angle (*F*_2, 126_ = 4.4, *p* = 0.014, partial η^2^ = 0.065), and no category × filming angle interaction (*F*_4, 126_ = 1.06, *p* = 0.38, partial η^2^ = 0.032). *Post-hoc* Tukey tests revealed that in the portrait and top videos, motion was identified earlier than in the left profile videos (*t*_126_ = −2.65, *p* = 0.024 and *t*_126_ = −2.47, *p* = 0.039, respectively), with no difference in onset between portrait and top videos (*t*_126_ = −0.18, *p* = 0.982).

When comparing the output of the frame-differencing procedure to the motion onset frames identified by a human observer, we found a large degree of convergence with the exact same motion onset frame identified by the two for 113 (83.7 %) videos. A close inspection of the motion onset detection in the remaining 22 videos revealed that the discrepancy was as a result of instances of oversensitivity of the frame-differencing procedure which resulted in detection of small shadows, wrinkles in clothing, or eye movements of the actor. To circumvent this issue, as well as the discovered undesirable systematic differences in motion onset, we decided to edit all videos by cropping out the excessive still frames in the beginning and assuring that humanly perceptible motion onset begins exactly at frame 2 for every video. The success of this editing was verified by passing the videos to the frame-differencing algorithm and a visual inspection for the second time, which were in agreement for all 135 videos. The scripts for performing the edits of the videos are made available. The edited videos in .m4v and .mat format can be found in the folder “Video Database” at https://osf.io/zexc4/.

### Category recognition

**Figure 6:**
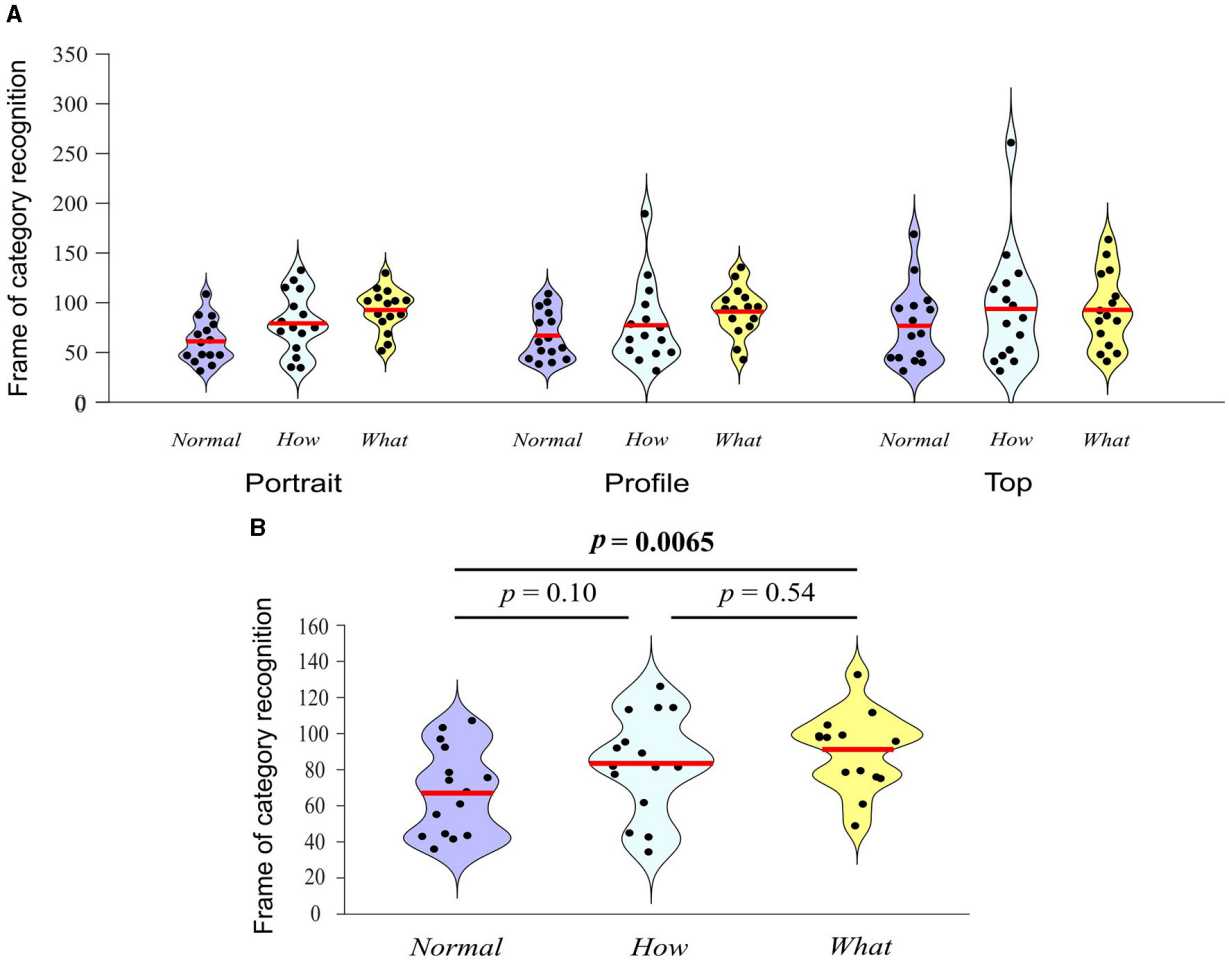
Distributions of the frames on which videos were recognized as *Normal, How*, or *What* for each category and filming angle **(A)** and Turkey tests for factor category **(B)**. Red lines indicate the respective means. In **(B)**, the outcome of Turkey tests comparing filming categories across filming angles is indicated above the violins.

In just 17 between-subject iterations, the staircase procedure converged on a single frame within each video of the original database. The convergence frame was in the range 31–259 frames (*Mean* = 81, *SD* = 35.73), corresponding to time 0.52–4.32 s (*Mean* = 1.35 s, *SD* = 0.6 s). The 2-way ANOVA investigating for differences in the timing of category recognition between videos from different category and filming angle revealed a significant effect of category (*F*_2, 126_ = 5.01, *p* = 0.008, partial η^2^ = 0.074), no effect of filming angle (*F*_2, 126_ = 0.95, *p* = 0.39, partial η^2^ = 0.015) and no category × filming angle interaction (*F*_2, 126_ = 0.41, *p* = 0.799, partial η^2^ = 0.013; Figure 6A). Figure 6B presents *post-hoc* Tukey tests on the factor category. These tests revealed that *Normal* videos were recognized on average 22.9 frames earlier than the *What* videos, with no significant difference between *Normal* and *How* and *How* and *What* videos.

This entire analysis investigating for differences in the timing of category recognition was repeated after the videos were edited to be equated for motion onset. The results of this analysis revealed a pattern of results identical to the one for the original video database. Each video's frame of category recognition, before and after editing is presented in Appendix A.

## Discussion

The present article introduces a novel database of 135 video stimuli for action observation research in neuroimaging settings. It includes high definition videos of a male actor performing object-oriented actions from three categories: kinematically natural and goal-intact *Normal* actions, kinematically unnatural and goal-intact *How* actions, and kinematically natural and goal-violating *What* actions. This publicly available dataset will facilitate the investigation of both bottom-up and top-down neural processing of the characteristics of observed human actions.

Psychometric evaluation of the videos indicated high video classification accuracy and inter-rater agreement, conceptually translating to good validity and substantial reliability of the video categories. Although classification accuracy was very high for videos from all categories, we identified that classification accuracy was consistently higher for *Normal* compared to *What* videos in the portrait, profile, and top filming angles. While statistically significant, this difference consisted of, on average, one additional video in 15 in the *What* category not being correctly classified, compared to the near perfect classification of *Normal* videos. Therefore, we refrain from interpreting it as a mental phenomenon of importance, or as a serious methodological challenge inherent in the use of our database. Instead, in line with the goal of the publication of our database, these findings could be the object of future psychophysics and neuroimaging investigations aimed at elucidating the recognition of different types of actions.

Subsequent to the psychometric evaluation, every video's exact frame of motion onset was identified with a custom-made motion detection system. Some undesirable differences in motion onset of the original video database as well as slight inaccuracies in the frame-tracing procedure were discovered, therefore we re-edited all videos to assure that perceptible motion begins at exactly the second frame in each video. This assures that the *Normal, How*, and *What* videos in each filming angle are equivalent in terms of motion onset, which facilitates their presentation in neuroimaging experiments and circumvents the issue of jittered triggering altogether.

Moreover, the frame on which each video becomes discernibly recognizable as *Normal, How*, or *What* was also identified with a novel up-down staircase procedure. We identified significant differences in timing of category recognition between the *Normal, How*, and *What* videos with the *Normal* actions recognized 20–25 frames (or 0.33 s−0.42 s) earlier than the *How* and *What* actions. Across videos, there were differences of up to ~220 frames (or 3.7 s) in the timing of action category recognition. These results further highlight the importance of incorporating video motion characterization procedures into future experimental designs. Identifying the respective onset frames allows for counteracting the described differences by incorporating appropriately timed triggers. For instance, in addition to sending a trigger at the moment of presentation of each video on the screen, the experimenters will be able to send a second trigger corresponding to the exact moment of recognition of each video as identified by the staircase procedures. As a cautionary note, given the discovered difference in recognition timing between *Normal* and *What* videos, this second trigger will tend to come on average 0.38 s later for the *What* videos. This implies that experimenters should carefully consider the length of their epoch to assure that their offset is within the presented video's duration for each condition. This way contrasts between *Normal* and *What* videos will be performed under identical action observation conditions without potential contamination from the video ending and the interstimulus interval. Although epoch length selection depends on the specific research question, commonly used time periods of 1 to 2.5 s can successfully be epoched following a category recognition trigger from each video from our database. This approach would then allow for more precise grand averaging of epochs, precisely timed to the actual onset of the characteristic of interest. It is expected that such an approach will improve the sensitivity of event-related neuroimaging experiments. For this reason, the video database, the psychometric and motion evaluations, and the scripts required for performing these evaluations are made freely available for future scientific use. Moreover, our two motion evaluation procedures are well suited for characterizations of other already existing (Cipriano et al., 2023; Di Crosta et al., 2020; Umla-Runge et al., 2012; Urgen et al., 2022) or to-be-developed sets of video stimuli. Furthermore, any additional evaluations of the videos such as collection of arousal and valence ratings, object familiarity ratings, imaginability ratings, normative validation in special populations, or collection of eye-tracking data are also greatly encouraged. Motor neuroscience studies of motor resonance or embodiment should particularly focus on characterizing the videos' imaginability, whereas cognitive neuroscience studies should focus on characterizing the videos for arousal and valence. Determining the extent to which participants are familiar with the depicted objects is equally relevant for both motor and cognitive experiments, especially with non-western participants. For a successful experimental design any potential differences along these dimensions should be controlled for. Another fruitful direction for future research is a validation of the results of our motion onset frame-differencing algorithm and our category recognition up-down staircase with more advanced artificial intelligence and marker less motion capture methods to assure their precision of capturing nuanced human movements.

A noteworthy limitation of our database is that our videos depict only a male actor. It is conceivable that nuanced gender effects could be present in investigations of motor resonance and embodiment, therefore, depending on the experimental design, future researchers may need to compare their outcome variable between male and female observers and if necessary supplement the video database with videos depicting a female actor. A further limitation is that in our videos filmed from profile orientation the actor is wearing different attire. This was done to provide better contrast between the actor and the background, however, it could potentially cause differences in the perception of these videos compared to the ones from the other angles. In spite of these limitations, our open-access video database greatly facilitates the process of stimulus preparation for subsequent action observation experiments. By relying on the present set of videos, researchers can design controlled investigations of the perception and comprehension of the kinematics of goal-directed human motion without investing resources into filming videos or depending on haphazard videos gleaned from the internet. Indeed, the reliance on standardized videos across experiments reduces the likelihood of introducing undesired variability into the experimental manipulation due to differences of stimuli. Therefore, reliance on the present set of videos could improve the comparability and replicability of action observation research within motor and cognitive neuroscience.

As it currently stands, the present dataset has a wide applicability for action observation research within various fields of inquiry. The videos were explicitly designed for application in electrophysiological (e.g., Electroencephalography and Magnetoencephalography), hemodynamic (functional Magnetic Resonance Imaging, Functional Near-Infrared Spectroscopy and Positron Emission Tomography), and transcranial stimulation (Transcranial Magnetic Stimulation, transcranial Direct Current Stimulation) studies of brain activity during action observation (e.g., Arnstein et al., 2011; Avanzini et al., 2012; Brunsdon et al., 2019; Dinomais et al., 2013; Nedelko et al., 2010). However, they can also be utilized in investigations of the neural encoding of the kinematics of observed action (Bourguignon et al., 2013; Marty et al., 2018; Savaki et al., 2022), the goals and intentions of observed actions (Hamilton and Grafton, 2006; Nicholson et al., 2017), as well as the actions and goals afforded by objects (Bach et al., 2011; Tipper et al., 2006). Since our videos are highly recognizable, they can also be used in investigations of all above mentioned phenomena during action imitation and action learning interventions for clinical populations with impaired action comprehension such as autism spectrum disorder (Kaokhieo et al., 2023) and schizophrenia (Enticott et al., 2008). Such interventions are expected to strengthen the action perception–execution association, facilitate action embodiment (Tschacher et al., 2017), and lead to improved social cognition. In addition, the videos from the *Normal* category can also serve as excellent training stimuli for action observation therapy—a promising intervention for motor recuperation in patients with movement disorders such as stroke (Ertelt et al., 2007; Fu et al., 2017) or Parkinson's disease (Pelosin et al., 2010) and an equally promising intervention for maintenance of motor ability and physical performance in healthy elderly (Rizzolatti et al., 2021). The videos can also be used as naturalistic visual stimuli for cognitive neuroscience experiments investigating, among others, attention to and memorization of human action, supplementing the frequently used static pictures and non-naturalistic point-light displays (Gao et al., 2015; Lu et al., 2016; Sifre et al., 2018). Further advances in all above-mentioned fields of research are necessary for gaining a more comprehensive understanding of the neural and cognitive processes which allow for perception and comprehension of observed action and, in turn, execution of appropriate corresponding motor reactions. Such understanding could foster training protocols for timely initial development and, in the case of acquired cognitive and motor impairments, effective recovery of these abilities—both crucial for the display of effective interpersonal behaviors within the cohesive structure of a healthy society.

## Conclusion

In closing, in hopes of facilitating action observation research in neuroimaging settings and encouraging open methodology, we provide a large open-access database of psychometrically evaluated videos of an actor performing movements explicitly designed to be kinematically correct or incorrect and goal-intact or goal-violating. We also provide the timing of action category recognition within each video for the sake of maximizing the precision of the assessment of the neural activity evoked by the observation of the different actions. The precise characterization of the videos in terms of psychometric properties and motion onset makes the present database a very suitable stimulus set for experiments aimed at investigating the neural correlates of perception, comprehension and acquisition of observed action.

## Data Availability

The datasets presented in this study can be found in online repositories. The names of the repository/repositories and accession number(s) can be found below: https://osf.io/zexc4/.

## Appendix

**Table A1:** Frame on which category is recognized for each video of the original database.

	***Normal***			***How***			***What***		
	**Portrait**	**Profile**	**Top**	**Portrait**	**Profile**	**Top**	**Portrait**	**Profile**	**Top**
Calculator	31	40	31	35	31	34	99	93	45
Cap	108	100	81	113	51	84	85	42	96
Case	62	44	97	115	48	67	129	80	84
Coffee jar	47	42	39	44	49	41	51	52	42
Comb	87	99	94	117	111	109	114	96	103
Cup	43	43	41	34	48	259	84	105	145
Earphone	59	82	93	74	66	102	111	95	89
Fork	86	64	169	132	98	148	105	132	160
Glasses	64	63	102	77	55	52	88	71	126
Hat	78	80	67	81	75	129	56	82	43
Hourglass	47	38	44	95	83	97	68	76	83
Mouse	35	54	43	44	42	40	88	93	53
Pen	47	51	66	74	78	113	102	103	130
Ruler	68	109	131	74	189	78	102	128	66
Scissors	48	89	45	71	127	46	103	111	79

**Table A2:** Frame on which category is recognized for each video of the edited database.

	***Normal***			***How***			***What***		
	**Portrait**	**Profile**	**Top**	**Portrait**	**Profile**	**Top**	**Portrait**	**Profile**	**Top**
Calculator	31	***24***	31	35	31	34	99	93	45
Cap	***94***	100	81	113	51	84	85	42	96
Case	62	44	97	115	48	67	129	80	84
Coffee jar	47	***40***	39	44	49	41	51	***51***	42
Comb	87	99	94	***114***	***104***	***102***	***110***	96	103
Cup	***38***	43	41	34	48	259	84	105	***140***
Earphone	59	82	***86***	74	66	102	111	95	89
Fork	86	64	***150***	132	98	***136***	105	***126***	160
Glasses	64	63	***63***	77	55	52	88	71	126
Hat	***74***	***74***	67	***45***	75	129	56	82	43
Hourglass	47	***37***	44	95	83	97	68	76	83
Mouse	35	***50***	43	44	42	40	***87***	93	53
Pen	47	51	***60***	74	***73***	***106***	102	***95***	***114***
Ruler	68	***105***	***110***	74	***187***	78	***98***	128	66
Scissors	48	89	45	71	***115***	46	***89***	111	79

Bold Italic indicates an edited video.

## References

[B1] Arnstein D. Cui F. Keysers C. Maurits N. M. Gazzola V. (2011). μ-suppression during action observation and execution correlates with BOLD in dorsal premotor, inferior parietal, and SI cortices. J. Neurosci. 31, 14243–14249. 10.1523/JNEUROSCI.0963-11.2011 21976509 PMC6623646

[B2] Avanzini P. Fabbri-Destro M. Dalla Volta R. Daprati E. Rizzolatti G. Cantalupo G. . (2012). The dynamics of sensorimotor cortical oscillations during the observation of hand movements: an EEG study. PLoS ONE 7:e37534. 10.1371/journal.pone.0037534 22624046 PMC3356327

[B3] Bach P. Bayliss A. P. Tipper S. P. (2011). The predictive mirror: interactions of mirror and affordance processes during action observation. Psychon. Bullet. Rev. 18, 171–176. 10.3758/s13423-010-0029-x 21327353 PMC3042113

[B4] Beattie G. Shovelton H. (2002). An experimental investigation of some properties of individual iconic gestures that mediate their communicative power. Br. J. Psychol. 526, 179–192. 10.1348/000712602162526 12031146

[B5] Biagi L. Cioni G. Fogassi L. Guzzetta A. Sgandurra G. Tosetti M. (2016). Action observation network in childhood: a comparative fMRI study with adults. Dev. Sci. 19, 1075–1086. 10.1111/desc.12353 26537750

[B6] Bourguignon M. De Tiège X. de Beeck M. O. Van Bogaert P. Goldman S. Jousmäki V. . (2013). Primary motor cortex and cerebellum are coupled with the kinematics of observed hand movements. Neuroimage 66, 500–507. 10.1016/j.neuroimage.2012.10.038 23108269

[B7] Braadbaart L. Williams J. H. G. Waiter G. D. (2013). Do mirror neuron areas mediate mu rhythm suppression during imitation and action observation?. Int. J. Psychophysiol. 89, 99–105. 10.1016/j.ijpsycho.2013.05.019 23756148

[B8] Bradski G. R. Kaehler A. (2008). Learning OpenCV: Computer Vision with the OpenCV Library. Sebastopol, CA: O'Reilly Media.

[B9] Brunsdon V. E. A. Bradford E. E. F. Ferguson H. J. (2019). Sensorimotor mu rhythm during action observation changes across the lifespan independently from social cognitive processes. Dev. Cogn. Neurosci. 38:100659. 10.1016/j.dcn.2019.100659 31132663 PMC6688050

[B10] Buccino G. Vogt S. Ritzl A. Fink G. R. Zilles K. Freund H. J. . (2004). Neural circuits underlying imitation learning of hand actions: an event-related fMRI study. Neuron 42, 323–334. 10.1016/S0896-6273(04)00181-3 15091346

[B11] Caillaud M. Bejanin A. Laisney M. Gagnepain P. Gaubert M. Viard A. . (2020). Influence of emotional complexity on the neural substrates of affective theory of mind. Hum. Brain Mapp. 41, 139–149. 10.1002/hbm.24794 31566290 PMC7267895

[B12] Cheng C.-H. Sun H.-H. Weng J.-Q. Tseng Y.-J. (2017). Differential motor cortex excitability during observation of normal and abnormal goal-directed movement patterns. Neurosci. Res. 123, 36–42. 10.1016/j.neures.2017.04.013 28457959

[B13] Cipriano M. Carneiro P. Albuquerque P. B. Pinheiro A. P. Lindner I. (2023). Stimuli in 3 Acts: a normative study on action-statements, action videos and object photos. Behav. Res. Methods 55, 3504–3512 10.3758/s13428-022-01972-8 36131196

[B14] Di Crosta A. Malva P. L. Manna C. Marin A. Palumbo R. Verrocchio M. C. . (2020). The Chieti Affective Action Videos database, a resource for the study of emotions in psychology. Sci. Data 7:32. 10.1038/s41597-020-0366-1 31964894 PMC6972777

[B15] Dinomais M. Lignon G. Chinier E. Richard I. Ter Minassian A. Tich S. N. (2013). Effect of observation of simple hand movement on brain activations in patients with unilateral cerebral palsy: an fMRI study. Res. Dev. Disabil. 34, 1928–1937. 10.1016/j.ridd.2013.03.020 23584173

[B16] Enticott P. G. Hoy K. E. Herring S. E. Johnston P. J. Daskalakis Z. J. Fitzgerald P. B. . (2008). Reduced motor facilitation during action observation in schizophrenia: a mirror neuron deficit? Schizophr. Res. 102, 116–121. 10.1016/j.schres.2008.04.001 18485674

[B17] Ertelt D. Small S. Solodkin A. Dettmers C. McNamara A. Binkofski F. . (2007). Action observation has a positive impact on rehabilitation of motor deficits after stroke. NeuroImage 36, T164–T173. 10.1016/j.neuroimage.2007.03.043 17499164

[B18] Fleiss J. L. (1971). Measuring nominal scale agreement among many raters. Psychol. Bull. 76, 378–382. 10.1037/h0031619

[B19] Fu J. Zeng M. Shen F. Cui Y. Zhu M. Gu X. . (2017). Effects of action observation therapy on upper extremity function, daily activities and motion evoked potential in cerebral infarction patients. Medicine 96:e8080. 10.1097/MD.0000000000008080 29049194 PMC5662360

[B20] Gao Z. Bentin S. Shen M. (2015). Rehearsing biological motion in working memory: an EEG study. J. Cogn. Neurosci. 27, 198–209. 10.1162/jocn_a_00687 25061930

[B21] Gera G. Freitas S. Latash M. Monahan K. Schöner G. Scholz J. (2010). Motor abundance contributes to resolving multiple kinematic task constraints. Motor Cont. 14, 83–115. 10.1123/mcj.14.1.83 20237405 PMC2843002

[B22] Hamilton A. F. Grafton S.T. (2006). Goal representation in human anterior intraparietal sulcus. J. Neurosci. 26, 1133–1137. 10.1523/JNEUROSCI.4551-05.2006 16436599 PMC6674581

[B23] Huettel S. A. (2012). Event-related fMRI in cognition. Neuroimage 62, 1152–1156. 10.1016/j.neuroimage.2011.08.113 21963919 PMC3277683

[B24] Iacoboni M. Molnar-Szakacs I. Gallese V. Buccino G. Mazziotta J. C. Rizzolatti G. (2005). Grasping the intentions of others with one's own mirror neuron system. PLoS Biol. 3:e79. 10.1371/journal.pbio.0030079 15736981 PMC1044835

[B25] Kaernbach C. (1991). Simple adaptive testing with the weighted up-down method. Perception and psychophysics 49, 227–229. 10.3758/BF03214307 2011460

[B26] Kaokhieo J. Tretriluxana J. Chaiyawat P. Siripornpanich V. Permpoonputtana K. Tretriluxana S. . (2023). Effects of repetitive transcranial magnetic stimulation combined with action-observation-execution on social interaction and communication in autism spectrum disorder: Feasibility study. Brain Res. 1804:148258. 10.1016/j.brainres.2023.148258 36702183

[B27] Levitt H. (1971). Transformed up-down methods in psychoacoustics. J. Acoust. Soc. Am. 49:467. 10.1121/1.1912375 5541744

[B28] Lu X. Huang J. Yi Y. Shen M. Weng X. Gao Z. (2016). Holding Biological Motion in Working Memory: An fMRI Study. Front. Hum. Neurosci. 10:251. 10.3389/fnhum.2016.00251 27313520 PMC4887503

[B29] Malfait N. Valyear K. F. Culham J. C. Anton J.-L. Brown L. E. Gribble P. L. (2010). fMRI activation during observation of others' reach errors. J. Cogn. Neurosci. 22, 1493–1503. 10.1162/jocn.2009.21281 19580392

[B30] Marty B. Bourguignon M. Jousmäki V. Wens V. Goldman S. De Tiège X. (2018). Movement kinematics dynamically modulates the Rolandic ~ 20-Hz rhythm during goal-directed executed and observed hand actions. Brain Topogr. 31, 566–576. 10.1007/s10548-018-0634-y 29445903

[B31] Moriguchi Y. Ohnishi T. Decety J. Hirakata M. Maeda M. Matsuda H. . (2009). The human mirror neuron system in a population with deficient self-awareness: an fMRI study in alexithymia. Hum. Brain Mapp. 30, 2063–2076. 10.1002/hbm.20653 18781590 PMC6871149

[B32] Muthukumaraswamy S. D. Johnson B. W. (2004). Primary motor cortex activation during action observation revealed by wavelet analysis of the EEG. Clini. Neurophysiol. 115, 1760–1766. 10.1016/j.clinph.2004.03.004 15261854

[B33] Nedelko V. Hassa T. Hamzei F. Weiller C. Binkofski F. Schoenfeld M. A. . (2010). Age-independent activation in areas of the mirror neuron system during action observation and action imagery. A fMRI study. Restorat. Neurol. Neurosci. 28, 737–747. 10.3233/RNN-2010-0542 21209489

[B34] Nicholson T. Roser M. Bach P. (2017). Understanding the goals of everyday instrumental actions is primarily linked to object, not motor-kinematic, information: evidence from fMRI. PLoS ONE 12:e0169700. 10.1371/journal.pone.0169700 28081175 PMC5231350

[B35] Orban G. A. Ferri S. Platonov A. (2019). The role of putative human anterior intraparietal sulcus area in observed manipulative action discrimination. Brain Behav. 9:e01226. 10.1002/brb3.1226 30740932 PMC6422812

[B36] Paxton A. Dale R. (2013). Frame-differencing methods for measuring bodily synchrony in conversation. Behav. Res. Methods 45, 329–343. 10.3758/s13428-012-0249-2 23055158

[B37] Pelosin E. Avanzino L. Bove M. Stramesi P. Nieuwboer A. Abbruzzese G. (2010). Action observation improves freezing of gait in patients with Parkinson's disease. Neurorehabil. Neural Repair 24, 746–752. 10.1177/1545968310368685 20453155

[B38] Platonov A. Orban G. A. (2016). Action observation: the less-explored part of higher-order vision. Sci. Rep. 6:36742 10.1038/srep36742 27857160 PMC5114682

[B39] Quandt L. C. Marshall P. J. (2014). The effect of action experience on sensorimotor EEG rhythms during action observation. Neuropsychologia 56, 401–408. 10.1016/j.neuropsychologia.2014.02.015 24568874 PMC4009369

[B40] R Core Team (2021). R: A Language and Environment for Statistical Computing. Vienna: R Foundation for Statistical Computing. Available at: https://www.R-project.org/

[B41] Ramseyer F. Tschacher W. (2011). Nonverbal synchrony in psychotherapy: coordinated body movement reflects relationship quality and outcome. J. Consult. Clin. Psychol. 79, 284–295. 10.1037/a0023419 21639608

[B42] Ramseyer F. T. (2020). Motion energy analysis (MEA): A primer on the assessment of motion from video. J. Couns. Psychol. 67, 536–549. 10.1037/cou0000407 32614233

[B43] Rizzolatti G. Fabbri-Destro M. Nuara A. Gatti R. Avanzini P. (2021). The role of mirror mechanism in the recovery, maintenance, and acquisition of motor abilities. Neurosci. Behav. Rev. 127, 404–423. 10.1016/j.neubiorev.2021.04.024 33910057

[B44] Rosenthal-von der Pütten A. M. Schulte F. P. Eimler S. C. Sobieraj S. Hoffmann L. Maderwald S. . (2014). Investigations on empathy towards humans and robots using fMRI. Comput. Hum. Behav. 33, 201–212. 10.1016/j.chb.2014.01.00430852995

[B45] Savaki H. E. Kavroulakis E. Papadaki E. Maris T. G. Simos P. G. (2022). Action observation responses are influenced by movement kinematics and target identity. Cereb. Cortex 32, 490–503. 10.1093/cercor/bhab225 34259867

[B46] Saxe R. Xiao D. K. Kovacs G. Perrett D. I. Kanwisher N. (2004). A region of right posterior superior temporal sulcus responds to observed intentional actions. Neuropsychologia 42, 1435–1446. 10.1016/j.neuropsychologia.2004.04.015 15246282

[B47] Scott M. W. Emerson J. R. Dixon J. Tayler M. A. Eaves D. L. (2020). Motor imagery during action observation enhances imitation of everyday rhythmical actions in children with and without developmental coordination disorder. Hum. Mov. Sci. 71:102620. 10.1016/j.humov.2020.102620 32452437

[B48] Sifre R. Olson L. Gillespie S. Klin A. Jones W. Shultz S. (2018). A longitudinal investigation of preferential attention to biological motion in 2- to 24-month-old infants. Sci. Rep. 8:2527. 10.1038/s41598-018-20808-0 29410484 PMC5802706

[B49] Sonkusare S. Breakspear M. Guo C. (2019). Naturalistic stimuli in neuroscience: critically acclaimed. Trends Cogn. Sci. 23, 699–714. 10.1016/j.tics.2019.05.004 31257145

[B50] Spengler S. Bird G. Brass M. (2010). Hyperimitation of actions is related to reduced understanding of others' minds in autism spectrum conditions. Biol. Psychiatry 68, 1148–1155. 10.1016/j.biopsych.2010.09.017 21130224

[B51] Stapel J. C. Hunnius S. van Elk M. Bekkering H. (2010). Motor activation during observation of unusual versus ordinary actions in infancy. Soc. Neurosci. 5, 451–460. 10.1080/17470919.2010.490667 20602285

[B52] Steen M. C. van der Steen M. C. Bongers R. M. (2011). Joint angle variability and co-variation in a reaching with a rod task. Exp. Brain Res. 2011, 411–422. 10.1007/s00221-010-2493-y 21127846 PMC3018264

[B53] Suzuki S. Abe K. (1985). Topological structural analysis of digitized binary images by border following. Comp. Vision, Graph. Image Proc. 30, 32–46. 10.1016/0734-189X(85)90016-7

[B54] Sylwester K. Lyons M. Buchanan C. Nettel D. Roberts G. (2012). The role of Theory of Mind in assessing cooperative intentions. Pers. Individ. Dif. 52, 113–117 10.1016/j.paid.2011.09.005

[B55] Tholen M. G. Trautwein F.-M. Böckler A. Singer T. Kanske P. (2020). Functional magnetic resonance imaging (fMRI) item analysis of empathy and theory of mind. Hum. Brain Mapp. 41, 2611–2628. 10.1002/hbm.24966 32115820 PMC7294056

[B56] Tipper S. P. Paul M. A. Hayes A. E. (2006). Vision-for-action: the effects of object property discrimination and action state on affordance compatibility effects. Psychonomic Bullet. Rev. 13, 493–498. 10.3758/BF03193875 17048736

[B57] Trettenbrein P. C. Zaccarella E. (2021). Controlling video stimuli in sign language and gesture research: the package for analyzing motion-tracking data. Front. Psychol. 12:628728. 10.3389/fpsyg.2021.628728 33679550 PMC7932993

[B58] Tschacher W. Giersch A. Friston K. (2017). Embodiment and schizophrenia: a review of implications and applications. Schizophr. Bull. 43, 745–753. 10.1093/schbul/sbw220 28338892 PMC5472128

[B59] Umla-Runge K. Zimmer H. D. Fu X. Wang L. (2012). An action video clip database rated for familiarity in China and Germany. Behav. Res. Meth. 44, 946–953. 10.3758/s13428-012-0189-x 22359253

[B60] Urgen B. A. Nizamoglu H. Eroglu A. Orban G. A. (2022). A large video set of natural human actions for visual and cognitive neuroscience studies and its validation with fMRI. Brain Sci. 13:61. 10.3390/brainsci13010061 36672043 PMC9856703

[B61] Urgen B. A. Orban G. A. (2021). The unique role of parietal cortex in action observation: functional organization for communicative and manipulative actions. Neuroimage 237:118220. 10.1016/j.neuroimage.2021.118220 34058335 PMC8285591

[B62] Vannuscorps G. Caramazza A. (2016). Typical action perception and interpretation without motor simulation. Proc. Natl. Acad. Sci. U.S.A. 113, 86–91. 10.1073/pnas.1516978112 26699468 PMC4711885

[B63] Vrigkas M. Nikou C. Kakadiaris I. A. (2015). A review of human activity recognition methods. Front. Robot. AI. 2:28. 10.3389/frobt.2015.00028

[B64] Wetherill G. B. Levitt H. (1965). Sequential estimation of points on a psychometric function. Br. J. Math. Stat. Psychol. 18, 1–10. 10.1111/j.2044-8317.1965.tb00689.x 14324842

[B65] Zouba N. Boulay B. Bremound F. Thonnat M. (2008). Monitoring activities of daily living (ADLs) of elderly based on 3D key human postures. ICVW. 2008, 37–50. 10.1007/978-3-540-92781-5_4

